# Supplementary material for: Integrating Dynamic 3D Chromatin Architecture and Gene Expression Alterations Reveal Heterosis in Brassica rapa
Source: Int J Mol Sci. 2024 Feb 22;25(5):2568. doi: 10.3390/ijms25052568 (PMC10931744; doi:10.3390/ijms25052568)
Supplement: Supplementary file 1 [file ijms-25-02568-s001.zip › Supplementary Figure. S1-S10.pdf]

Supplementary Figures

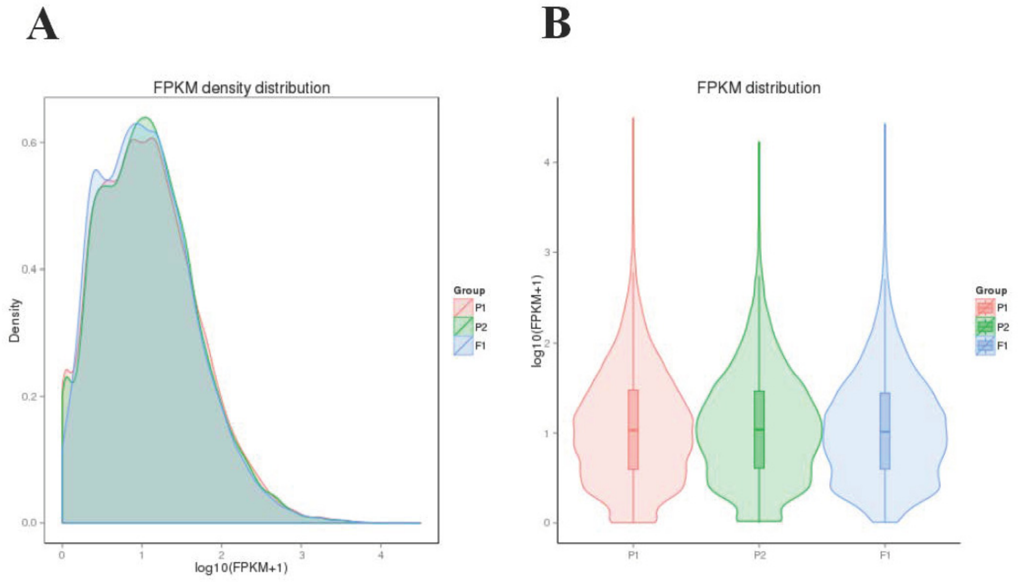

Figure S1 Genome wide expression profile of the w30(P2)-/082(P1)-/F_1 traid.

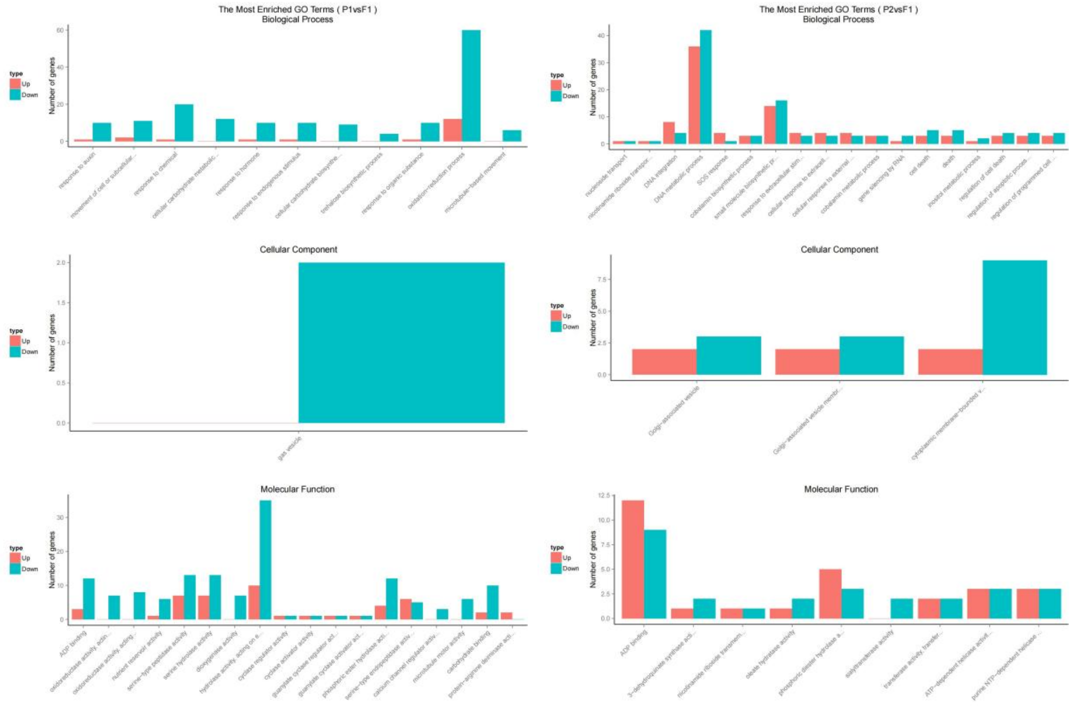

Figure S2 The most enriched GO terms with up-regulated DEGs and down-regulated DEGs.

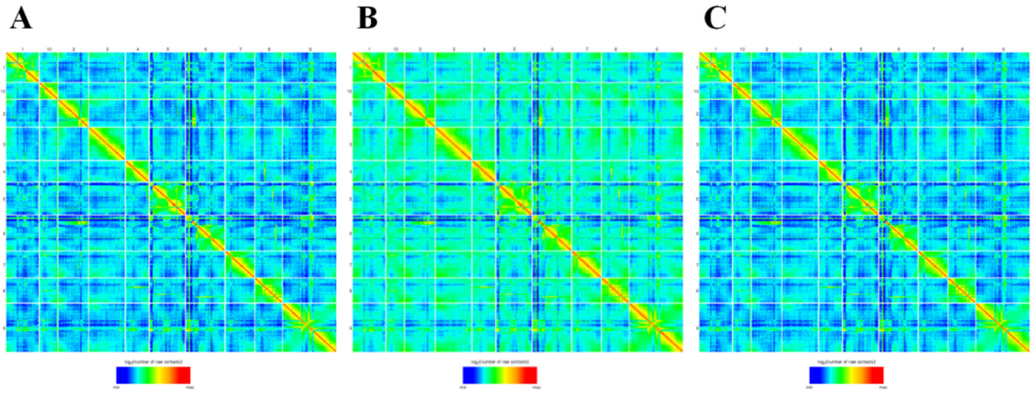

Figure S3 Genome-wide Hi-C contact maps for 082, w30 and F_1 at 1 Mb resolution.

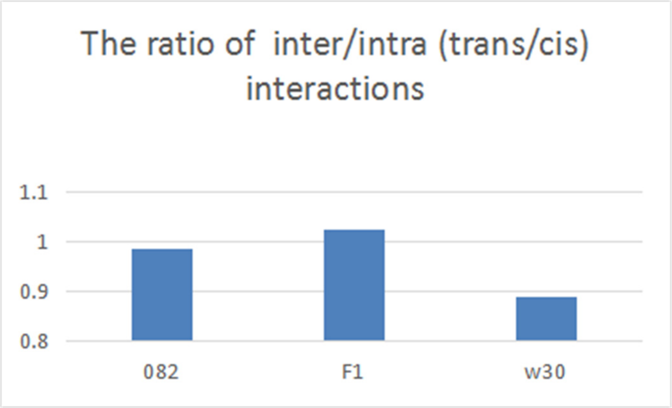

Figure S4 Ratio of inter/intra interactions in 082, w30 and F_1.

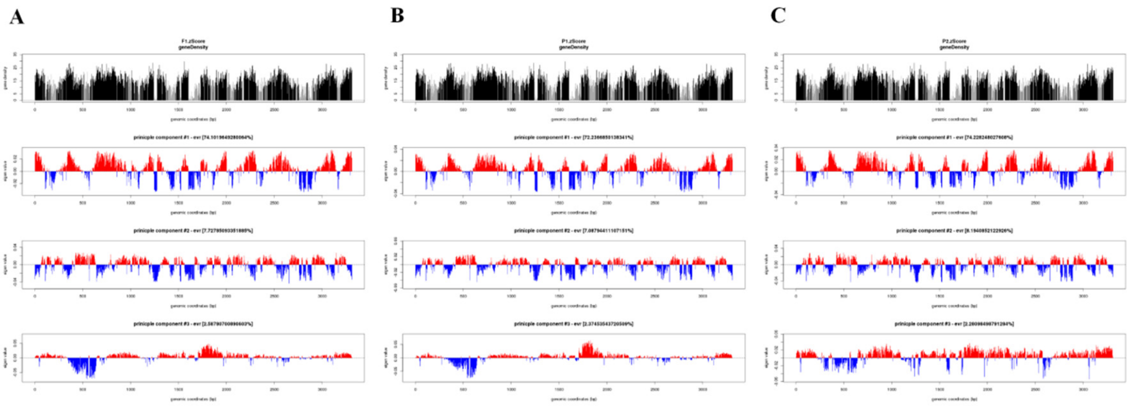

Figure S5 Gene density distribution under different genomic coordinates (bp) with the 1st (2nd, 3rd) principal component. With the y-axis 0 scale line as the reference, above is Compartment A and below is Compartment B.

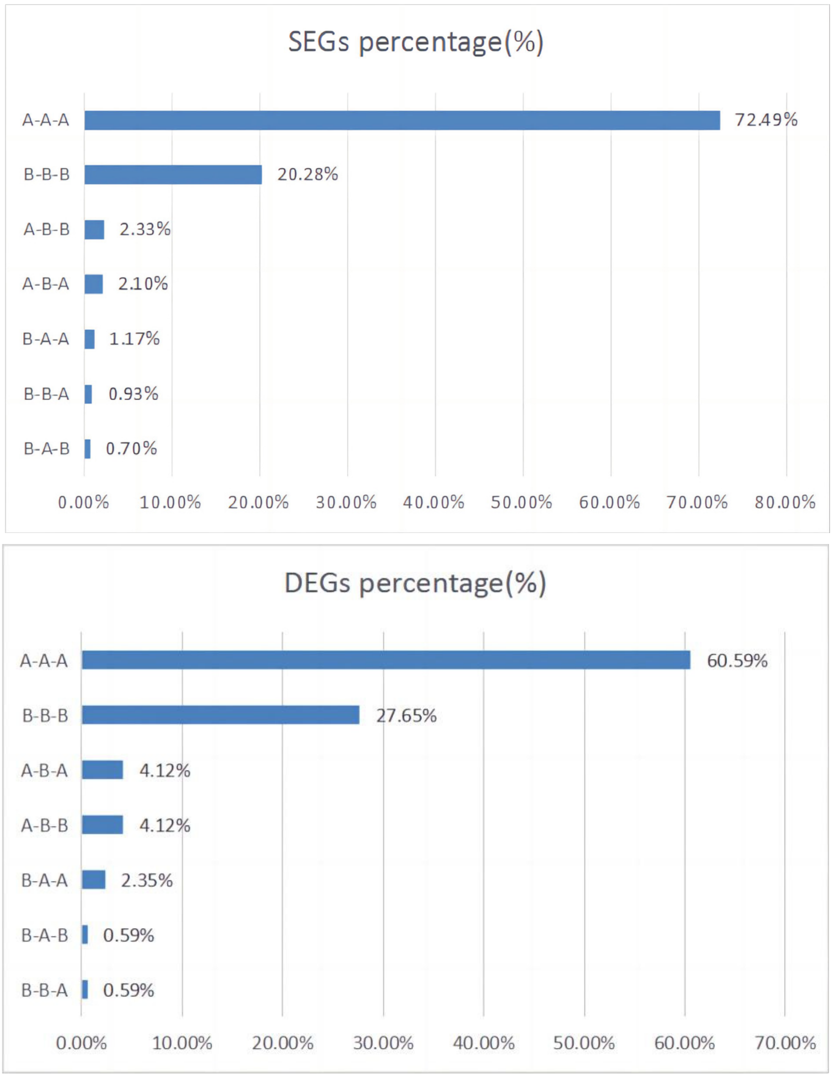

Figure S6 The percentage of the A/B compartments shift of SEGs and 296 DEGs.

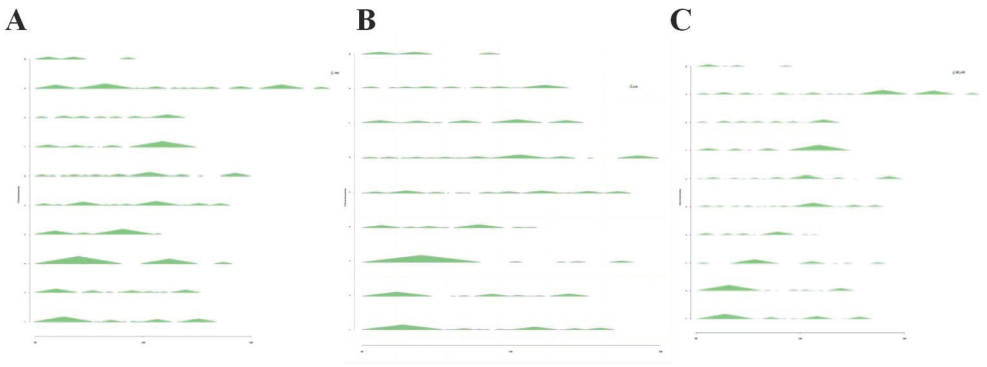

Figure S7 The distribution of TAD genome.

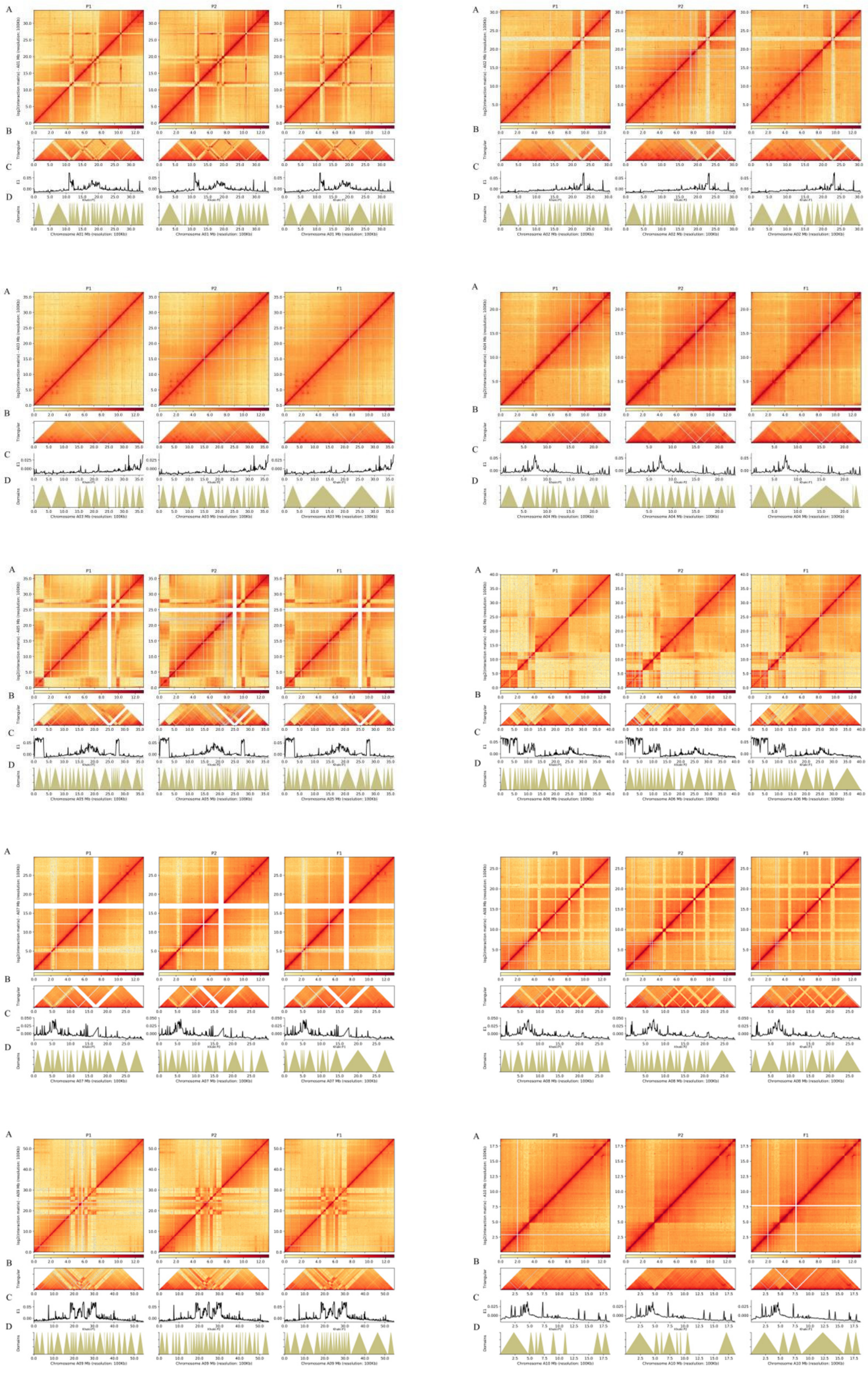

Figure S8 Analysis of 10 pairs of intra-chromosome interactions of the F_1 and its parents' samples.

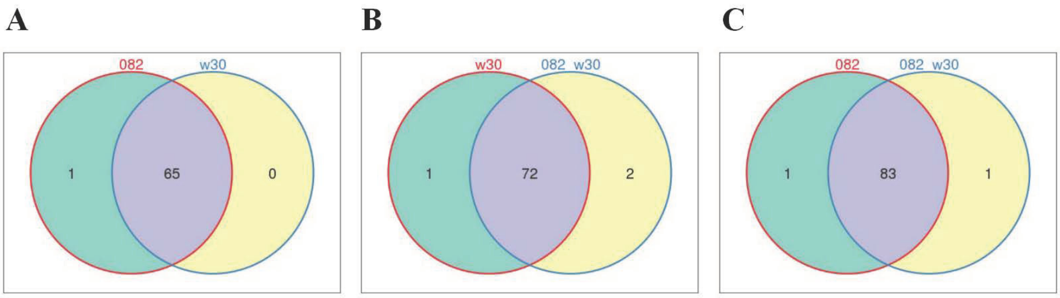

Figure S9 Indicated F_1 and its parents' Venn diagrams of the TAD boundary.

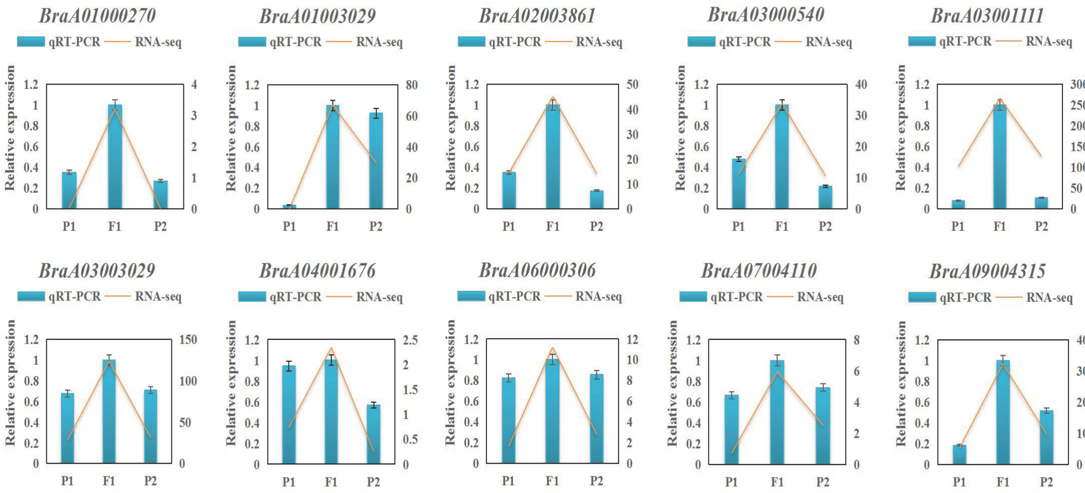

Figure S10 qRT-PCR verified 10 DEGs between the parents and F_1.
